# Experiences of Teachers and Occupational Therapists in Identifying Activity Problems Among Primary School Students With Developmental Difficulties in Indonesia: A Focus Group Interview

**DOI:** 10.1155/oti/4812427

**Published:** 2026-07-28

**Authors:** Putri Ashari, Cahya Ramadani Renhoran, Dwi Ayu Nur Komariyah, Yu Ishibashi

**Affiliations:** ^1^ Department of Occupational Therapy, Tokyo Metropolitan University, Tokyo, Japan, tmu.ac.jp; ^2^ Occupational Therapy Study Program, Vocational Higher Education Program, Universitas Indonesia, Depok, Indonesia, ui.ac.id

**Keywords:** activity problem, developmental difficulties, primary school, school-based occupational therapy, shared perspective

## Abstract

**Background:**

An interprofessional approach integrating the perspectives of teachers and school‐based occupational therapists is crucial for identifying and addressing complex activity problems among students with developmental difficulties. Nonetheless, the process through which professionals jointly perceive and describe these problems remains underexplored in Indonesia.

**Objective:**

This study is aimed at describing the experiences of Indonesian teachers and occupational therapists in identifying activity problems among primary school students with developmental difficulties.

**Methods:**

This descriptive qualitative study included 15 participants (seven teachers, one assistant teacher, and seven occupational therapists) who were purposively sampled and invited to participate in four online focus group interviews. Data were analyzed using inductive content analysis supported by member checking, peer debriefing, and an audit trail to ensure trustworthiness.

**Results:**

Two primary descriptive themes were identified as follows: (i) “holistic approaches to the management of student activity problems” and (ii) “students′ functional participation challenges at school.” The participants perceived those restrictions on students′ participation in expected activities resulted from the dynamic interplay between student capacities and contextual environmental constraints.

**Conclusions:**

These findings underscore the value of integrating diverse professional perspectives to capture the complex, transactional nature of students′ functional participation challenges in educational environments. School‐based occupational therapy practices should adopt holistic, collaborative approaches that empower school personnel and align with each school′s context. Further studies are necessary to develop contextually relevant assessments, protocols, and support programs addressing participation restrictions in Indonesian school settings.

## 1. Introduction

Developmental difficulties refer to signs or symptoms of arrest, alteration, or delay in one or more developmental domains in the absence of a specific disorder or disability [1]. Globally, an increasing number of children experiencing developmental difficulties enter mainstream primary schools with varying levels of functional readiness for academic tasks and other complex school‐based occupations [2]. These differences are often subtle and not immediately observable; consequently, teachers may struggle to identify them accurately and sometimes misinterpret functional variations as behavioral problems [3]. Such misinterpretation may lead to a mismatch between individual student capacities and the demands of school tasks, thereby restricting their ability to meet school expectations and increasing the risk of delayed intervention or inappropriate classroom support.

Student activity problems can be understood more comprehensively using the International Classification of Functioning, Disability, and Health—Child and Youth Version (ICF‐CY) framework, which links students′ conditions to contextual factors [4, 5]. Within the ICF‐CY framework, successful early identification largely depends on teachers′ ability to recognize subtle developmental difficulties and early indicators of occupational performance problems, given their continuous observation of students′ daily school routines. Alali et al. [6] highlighted the importance of teachers′ knowledge of the common indicators of developmental difficulties for facilitating early identification in educational settings. However, substantial disparities persist in how Indonesian teachers identify and support students with less visible developmental difficulties [7, 8]. These students may remain under‐recognized in mainstream classrooms despite experiencing functional and academic challenges [9, 10]. This inconsistency reflects a structural gap in inclusive education characterized by a mismatch between classroom demands and teachers′ knowledge and preparedness, underscoring the need for strengthened support systems and interdisciplinary collaboration.

In Indonesia, occupational therapists (OTs) are strategically positioned to bridge the gap in inclusive education. National policy mandates that OTs contribute to inclusive education by providing school‐based services that support student participation in academic, social, and self‐care activities [11]. This mandate aligns with the Occupational Therapy Practice Framework (OTPF), which emphasizes the role of OTs in holistically evaluating students′ occupational performance to facilitate effective collaboration with teachers and other professionals, thereby supporting students′ full participation [12, 13]. Despite this policy direction, existing evidence suggests that teachers often struggle to communicate their classroom observations, particularly regarding classroom expectations and contextual constraints, to OTs [14]. In Indonesia, systemic barriers, such as a critical shortage of practitioners, amplify these challenges. Data from the Indonesian Health Council reported 2395[15] registered OTs, highlighting a substantial gap between workforce capacity and service demand. This scarcity has led to a predominance of indirect or clinic‐based service models, limiting opportunities for OTs to directly observe and understand students′ occupational problems in their natural school environments.

Consequently, the identification of activity problems faced by students with developmental difficulties remains fragmented across the Indonesian educational system. Disparities in teachers′ ability to recognize subtle difficulties, communication barriers between educators and OTs, and limited availability of school‐based OT services collectively create a significant gap in effective identification and support. To address this gap, the present study is aimed at describing the shared experiences of teachers and OTs in identifying activity problems among primary school students with developmental difficulties in Indonesia. By exploring these professional perspectives, this study provides foundational insights to inform future research and the development of collaborative interventions and school‐based support programs that optimize student participation and educational outcomes.

## 2. Methods

### 2.1. Study Design

This descriptive qualitative study sought to explore the participants′ experiences and perspectives, generating grounded, authentic insights that reflect their reality [16]. Online focus group interviews (FGIs) were conducted with teachers and OTs to gain in‐depth insights into their understanding and experiences of identifying activity problems among primary school students with developmental difficulties. FGIs are widely recognized for their high face validity and ability to capture diverse viewpoints through dynamic interactions among group members [17]. Notably, the online format enabled the researchers to overcome geographical barriers across the Indonesian archipelago and accommodate participants′ convenient schedules [18].

### 2.2. Ethical Considerations

This study was approved by the Research Ethics Committee of Tokyo Metropolitan University (Approval No. 24099). Prior to data collection, written informed consent was obtained from all participants after they were comprehensively informed of the study′s purpose, procedures, and potential risks and benefits. Participant confidentiality was strictly maintained by securely storing and analyzing research data at Tokyo Metropolitan University using coded identifiers. Digital data were stored on password‐protected hard drives, and no personally identifiable information appeared in publications or presentations. In accordance with institutional guidelines, both digital and physical materials will be permanently disposed of 3 years following publication.

### 2.3. Participants

The research was conducted from March to May 2025. Participants were recruited through purposive sampling via a research recruitment poster shared on social media, which invited interested individuals to complete a Google Form. The inclusion criteria were as follows: (i) having at least 3 years of experience working as registered OTs, classroom teachers, teacher assistants, or shadow teachers in a primary school setting, and (ii) having direct experience working with students with developmental difficulties.

Students with developmental difficulties were characterized as those demonstrating at least one indicator across the following domains: sensory modulation (e.g., constant shifting from their seats); visual‐spatial perception (e.g., difficulty distinguishing or naming size, position, geometric shapes, and basic colors); cognitive and executive functions (e.g., frequent forgetfulness, poor attention span, rapid loss of newly learned material); social communication behavior (e.g., limited eye contact, atypical use of toys or playing with toys in ways different from their intended functions, restricted social interactions); and preacademic skills (e.g., confusion of similar letter forms, such as b/d, u/y, and l/i, and difficulties with numbers and letters). These indicators were drawn from common characteristics associated with developmental difficulties identified in a previous study on teachers′ knowledge [6]. In this study, the indicators were not diagnostic markers of a clinical disorder; rather, they served as observable signals of developmental difficulties in school settings. Therefore, these indicators do not represent a clinically unified diagnostic category but are operationalized as a functional umbrella construct for school‐based observation.

The primary researcher contacted prospective participants who met the inclusion criteria to confirm their eligibility and availability and to obtain informed consent. Those who agreed to participate were assigned to one of the four focus groups, organized by years of work experience to facilitate comfortable interaction among the participants, mitigate power imbalances, and support balanced group discussions. This setting allowed all participants to share their experiences comfortably, irrespective of their specific discipline.

### 2.4. Data Collection

Online FGIs were conducted via the Zoom platform on four occasions between April and May 2025. During the sessions, participants were encouraged to turn their cameras on to foster natural, active interactions and enable the observation of nonverbal communication [19]. A total of 15 participants (seven OTs, seven classroom teachers, and one assistant teacher) were divided into four focus groups. Consistent with methodological guidance indicating that three to four focus groups are sufficient to reach data saturation, each group comprised three to four participants to promote active engagement in the online format and to ensure all participants had sufficient time to speak [18, 20]. Because online FGIs may affect participants′ well‐being due to screen fatigue, each session was limited to 90–120 min, and participants were permitted to request a short break as needed. Each group included both OTs and teachers to ensure a variety of professional perspectives on student activity problems. This composition allowed for a critical dialogue that reflected real‐world school dynamics, generating rich contextual data by capturing the interactive and transactional identification processes across both disciplines.

Discussions were guided by a semistructured guide previously developed and piloted to ensure the questions were clear and aligned with the research aims. Five open‐ended guiding questions were used to explore the participants′ experiences, perspectives, and insights regarding the identification of activity problems among primary school students with developmental difficulties (Table 1). The primary researcher (P.A.), a master′s student in occupational therapy with professional experience working with school‐age children in healthcare and educational settings and prior experience observing several online FGIs, moderated each session. Two other researchers (C.R.R. and D.A.N.K.), who both hold master′s degrees in occupational therapy and currently work in pediatric practice, alternately acted as assistant moderators and observers. Immediately after each session, the research team held a debriefing to compare notes, highlight key points, and discuss group dynamics and emerging ideas. This process supported reflexivity and functioned as a verification step that minimized bias [20].

**Table 1:** Semistructured focus group interview guide.

No	Questions
1.	What is the first thing that comes to mind when you hear the term “a student with developmental difficulties”?
2.	Reflecting on your initial experience working with a student with developmental difficulties. What makes you notice that she/he might be experiencing developmental difficulties?
3.	Based on your daily observation, what specific academic‐related task problems do these students demonstrate in school?
4.	Based on your daily observation, what functional performance problem do these students demonstrate in school?
5.	If you were asked to mention the single most common problem in the classroom activity for students with developmental difficulties, what would it be?

Each session lasted 77–100 min and was recorded in both video and audio formats. Recordings and field notes were transcribed using the Notta transcription software, then authenticated through repeated listening, verification, and manual correction to ensure accuracy. Participants were assigned numerical codes throughout the transcription, analysis, and reporting processes to protect their confidentiality. Finally, verbatim transcripts were sent to participants as part of the transcript verification process to ensure the accuracy and credibility of the data before analysis. Participants provided clear feedback, including additional illustrative examples and clarifications of specific information. These additions were treated analytically as elaborative clarifications of material already discussed. Consequently, their input was incorporated into the verified transcripts prior to the inductive content analysis process.

### 2.5. Data Analysis

Given that previous studies on the topic are limited and existing knowledge remains fragmented, inductive content analysis was conducted to describe the phenomenon of interest [21]. Inductive content analysis is a data‐driven process in which researchers identify patterns in data and organize them into categories and broader themes at various levels of abstraction and interpretation [22]. The analysis followed three main phases: preparation, organization, and reporting [23].

In the preparation phase, all verbatim interview transcripts (four documents) were read repeatedly to develop an immersive, comprehensive understanding of the data. Three researchers (P.A., C.R.R., and D.A.N.K.) independently identified meaningful units related to the participants′ experiences in identifying activity problems among primary school students with developmental difficulties. A total of 334 meaning units were selected for subsequent analysis.

**Figure 1:**
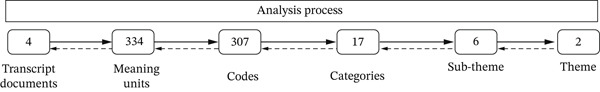
Analysis process.

In the organization phase, the primary researcher (P.A.) initially coded the selected meaning units, yielding 307 initial codes. The other researchers (C.R.R. and D.A.N.K.) then reviewed and confirmed the coding decisions to enhance analytical consistency and credibility. Through an iterative process of constant comparison, codes with similar semantic meanings were grouped into distinct categories. These emerging categories were further divided into subthemes and overarching themes (Figure 1). The descriptive themes achieved saturation when no new codes or categories emerged from the data, and the existing categories were densely supported with participants′ excerpts. Disagreements were resolved through detailed reflexivity and discussion until the entire research team (C.R.R., D.A.N.K., and Y.I.) reached a consensus, ensuring intercoder agreement and analytical consistency.

For example, the meaning unit, “Although the instructions are simple, there must be repetition… sometimes the second instruction is missed,” was coded as “difficulty processing multi‐step instructions.” Subsequently, this code was grouped under the category “cognitive processing and information retention,” contributing to the subtheme “students′ internal barriers”. It was ultimately integrated into the overarching theme “students′ functional participation challenges at school.”

Throughout the analysis, an audit trail was maintained by retaining multiple versions of coding matrices, category structures, and theme developments to document all methodological and analytical decisions over time. In the reporting phase, findings were presented using a transparent analytical process. Once the inductive content analysis was complete, member checking was conducted with all participants by sharing the final synthesized themes, subcategories, and categories, including supporting meaning units. Participants validated that the findings accurately reflected their experiences, and no changes were needed to the themes, categories, or subcategories, thereby enhancing the credibility and trustworthiness of the study. This study was reported in accordance with the Standards for Reporting Qualitative Research (SRQR) guidelines [24].

## 3. Results

Fifteen participants (seven OTs, seven classroom teachers, and one assistant teacher) with professional experience ranging from 3 to 27 (median = 8 years) were included in this study. Most participants were employed in private schools (*n* = 13), with a few working in public schools (*n* = 2). All participants worked on Java island, including Jakarta, West Java, Banten, Central Java, and Yogyakarta. Across these settings, the participants reported working with students who exhibited a range of characteristics of developmental difficulty. The most frequently observed characteristics were limited social interaction (*n* = 13), followed closely by forgetfulness accompanied by a short attention span and challenges retaining newly learned material (*n* = 12). Fewer participants encountered students who struggled to name basic colors and geometric shapes (*n* = 5) or who played with toys in ways that did not align with their intended function (*n* = 4). The demographic characteristics of the participants are summarized in Table 2.

**Table 2:** Demographic characteristics of participants.

Participant		Profession	Work experience in the current school	Total work experience	School type	Location
FGI Group 4	1	Classroom teacher	3 years	4 years	Private	Depok, West Java
	2	Classroom teacher	3 years	3 years	Private	West Jakarta, Jakarta
	3	OT	5 years	10 years	Private	East Jakarta, Jakarta
	4	OT	1 and 6 months	9 years	Private	South Jakarta, Jakarta

FGI Group 3	1	Assistant teacher	8 years	8 years	Private	Depok, West Java
	2	Classroom teacher	4 years	4 years	Public	Semarang, Central Java
	3	OT	6 years and 8 months	7 years	Private	South Jakarta, Jakarta

FGI Group 2	1	Classroom teacher	5 years and 10 months	11 years	Private	South Tangerang, Banten
	2	Classroom teacher	5 years	5 years	Private	Depok, West Java
	3	OT	10 years	10 years	Private	East Jakarta, Jakarta
	4	OT	14 years	22 years	Private	South Jakarta, Jakarta

FGI Group 1	1	Classroom teacher	27 years	27 years	Private	Bogor, West Java
	2	Classroom teacher	9 years	20 years	Public	Bantul, Yogyakarta
	3	OT	22 years	22 years	Private	South Jakarta, Jakarta
	4	OT	16 years	24 years	Private	Depok, West Java

Inductive content analysis yielded two primary themes that captured the participants′ shared experiences and interpretations: (i) “holistic approaches to the management of student activity problems” and (ii) “students′ functional participation challenges at school.” The former reflected how OTs and teachers identify and address activity‐related challenges while considering several interacting factors among students, school tasks, and classroom environments. Meanwhile, the latter highlighted the range of difficulties students experienced in daily classroom tasks, routines, and interactions. Table 3 provides a comprehensive overview of the themes, subthemes, and categories that comprise these analyses.

**Table 3:** Details of themes, subthemes, and categories.

Theme	Subtheme	Category
Holistic approaches to the management of student activity problems	Shared awareness and understanding	Integration of pedagogical and clinical lenses
		Collective responsibility regarding student problems
	Systematic evidence‐based identification process	Structured identification process
		Longitudinal and ecological validation
	Comprehensive support provision strategy	Instructional and environmental adaptation
		Integrative coordination for effective intervention

Students′ functional participation challenges at school	Students′ internal barriers	Cognitive processing and information retention
		Sensory processing sensitivities
		Physical coordination and motoric delays
		Social–emotional and behavior regulation
	Contextual and environmental constraints	Peer social dynamic and exclusion
		Home‐school factor problems
	Students′ restrictions on expected activities	Educational activity challenges
		Daily living and self‐care independence
		Spiritual and cultural participation

### 3.1. Theme 1: Holistic Approaches to the Management of Student Activity Problems

These FGIs explored participants′ experiences with identifying activity problems among students with developmental difficulties. The participants described how they recognized, interpreted, and applied clinical and pedagogical information to develop integrated collaborative support systems within schools. This process reflected a holistic approach where distinct professional perspectives synchronized over time, shifting service delivery from isolated, single‐profession management to multiple‐stakeholder interventions. This collaborative framework comprises three interconnected components: (i) shared awareness and understanding, (ii) a systematic, evidence‐based identification process, and (iii) a comprehensive support provision strategy, all of which are detailed below.

#### 3.1.1. Shared Awareness and Understanding

The participants emphasized that identifying activity problems requires heightened awareness among all school professionals and a shared sense of responsibility. This collective understanding emerged from integrating two distinct yet complementary lenses to detect early signs of difficulty.

From a pedagogical perspective, teachers highlighted academic achievement and classroom engagement as the primary indicators of potential challenges:

As a classroom teacher, second‐grade students are generally expected… If a student struggles across several subjects, that becomes a warning sign for me. (P10)

In contrast, OTs described student difficulties through a functional and clinical lens that looks beyond the grade book, focusing particularly on students′ interaction with the environment. They analyzed developmental patterns and occupational performance as the foundational pillars of their professional reasoning:

We look at how students behave and how they relate to their environment, especially when they are playing with peers or attempting to communicate with others. (P14)

As occupational therapists, our first step is to observe… we examine developmental patterns, including how the student plays, writes, and sits before beginning the clinical reasoning process (P3)

Importantly, the role of collective responsibility in addressing student problems was highlighted by the emotional challenges teachers faced in navigating inclusive classroom environments. One teacher reflected on the anxiety and uncertainty felt prior to establishing a shared interprofessional understanding:

I was concerned …. I was worried about how to support his learning… At that time, I felt anxious and could not imagine how the teaching sessions would unfold. (P13)

#### 3.1.2. Systematic Evidence‐Based Identification Process

The participants delineated a structured, stepwise process for identifying student‐specific needs, evaluating developmental readiness, and analyzing functional issues. This sequential pathway typically commenced at school admission and extended throughout the academic year:

There are several stages. We begin with a classical assessment and then proceed to individual assessments. During pre‐registration, I ask parents about the child′s developmental history. Because we are a private school that provides inclusive services, we consistently follow this process. (P4)

OTs and teachers emphasized the necessity of ongoing and repeated observation to ensure the consistency and ecological validity of the identified signs across diverse school contexts. This longitudinal approach prevented misidentification, ensuring that student challenges were interpreted authentically within their immediate classroom context:

Based on my experience, I always start with observation. I observed the student for one full day, both inside and outside the classroom, and then continued observing for about three to four months. After that, we discuss the observations with the classroom teacher to see whether they noticed the same signs. (P7)

Additionally, the participants described a continuous framework for monitoring, evaluation, and documentation to track academic and functional trajectories. This data‐driven process served as a critical foundation for professional referrals and structured consultations with parents, ensuring that decisions were based on persistent patterns rather than subjective impressions:

Each grade level has specific targets, so the first thing we consider is whether the student is meeting those targets. Second, we assess the student′s social skills with peers, which are monitored for approximately three to six months. Later, during a meeting with the parents, these findings are discussed. I would then request that the principal refer the student to a professional if the signs persist after six months (P8)

#### 3.1.3. Comprehensive Support Provision Strategy

The participants expressed that, upon identifying specific student needs, collaborative efforts among teachers, therapists, and school staff were essential in designing personalized support.

They described planning, tailoring, and modifying classroom environments, tasks, and learning strategies to support students′ participation in academic and social activities. Furthermore, they emphasized the critical importance of temporal and instructional adjustments for these students:

In terms of academics… we extend their time while the other students already go home. For reading, because of short‐term memory difficulties, we called them every hour to read for three or five minutes… Those are the adjustments we make for academic purposes. (P1)

Spatial settings and proximity to the teacher served as essential environmental strategies for providing immediate support in public school settings:

When it comes to academics, the point is to give the student tasks tailored to their ability. Moreover, they usually sit right in front of me so that I can stay close to them. Since this student really needs special assistance. (P2)

The participants also emphasized that continuous communication and collaboration among all stakeholders were crucial for ensuring sustainable and appropriate follow‐up. This collaborative network actively involved teachers, parents, school principals, counselors, OTs, and other school professionals:

We still need confirmation from the classroom teacher. After that, we proceed to the assessor or psychologist at the school and invite the parents to a follow‐up intervention plan. Our school has a therapy center, so we can directly refer the students there. (P7)

### 3.2. Theme 2: Students′ Functional Participation Challenges at School

These FGIs explored the participants′ experiences in managing the multifaceted challenges faced by students with developmental difficulties. The data identified diverse barriers that hindered students′ independent and effective participation in the school environment, highlighting a complex interplay between individual student capacities, external environmental conditions, and specific curricular demands. Consequently, three subthemes emerged from the analysis: (i) students′ internal barriers, (ii) contextual and environmental constraints, and (iii) students′ restrictions on expected activities. The details of these difficulties are as follows:

#### 3.2.1. Students′ Internal Barriers

The participants reported that internal factors, such as information processing, sensory sensitivities, behavioral regulation, and motor coordination, profoundly influenced students′ school engagement.

Specifically, limitations in cognitive processing and executive functioning were identified as primary barriers affecting daily classroom performance:

Even simple instructions must be repeated. When there are only two simple instructions, such as ‘take a pencil and then take a piece of paper’, the second instruction is sometimes missed. (P13)

The participants noted that some students exhibited sensory sensitivities, which created a substantial barrier to classroom engagement, often leading to the avoidance of specific tasks:

He tends to avoid things that are too noisy, or situations related to temperature sensitivity. Certain materials are not suitable for him, either because of texture or other tactile challenges. (P9)

OTs and teachers reported that students exhibited significant motor‐skill challenges that impacted performance relative to age‐appropriate expectations. Given that proficiency in this domain is foundational for functional mobility, these difficulties directly hindered students′ active participation in physical education:

Kicking the ball is also a problem. By second grade, students are expected to have already mastered kicking. He still wobbles when kicking the ball and has difficulties when running. (P8)

Furthermore, challenges with social‐emotional and behavioral regulation were commonly reported. The participants described students′ inability to maintain a stable emotional state, noting that exaggerated emotional responses frequently disrupted their social dynamics:

His tone of voice is sometimes uncontrollable and overly emotional with his friends. In situations that seem normal to other children, his emotions appear exaggerated. (P6)

This lack of regulation manifested as student difficulties in maintaining task persistence, attention, and social communication during group activities.

The student really struggles, whether with communicating or even something as simple as staying in a group… the student still needs a lot of movement; after sitting for only a short time, the student runs off and cannot stay for long. (P11)

Some difficulties are related to eye contact, attention during conversation, behavior, and similar issues. (P4)

#### 3.2.2. Contextual and Environmental Constraints

The participants reported that environmental barriers within the social and physical classroom environments shape students′ performance and participation at school.

Peer relationships emerged as a prominent concern, with both OTs and teachers noting that these students frequently faced social exclusion, neglect, or being avoided by their classmates:

The response from their friends was often neglectful. Peers expressed attitudes such as, ‘Well, you can do whatever you want… as long as I finish mine…’ Avoidance was also exhibited, with peers stating, ‘Oh, I do not want to be with this student because he cannot do the assignment.’ (P11)

Beyond the classroom, the participants described school functioning as inseparable from home routines, including emotional regulation and lifestyle habits, which were perceived to impact students′ overall school readiness and engagement directly:

Sleep schedules are highly influential, and some students show explosive emotions linked to poor sleep schedules. Rest and sleep really need to be regulated. The same applies to nutrition; the food these students consume affects them as well. Everything has an impact on how they function while studying at school. (P9)

For example, when conflicts arise during social interactions, they may only respond by shouting or getting angry. This is because many of them spend a lot of time watching television at home and using gadgets as well… (P7)

#### 3.2.3. Students′ Restrictions on Expected Activities

The participants described how students with developmental difficulties encountered substantial challenges in fulfilling their occupational roles as learners. These barriers manifested as functional gaps in meeting the school community′s academic, personal, and cultural expectations.

Within the academic domain, students′ performance frequently fell short of grade‐level expectations; for instance, by the second or third grade, many students still struggled with basic comprehension:

Children suspected of having developmental difficulties often continue to struggle in the second or third grade. They have difficulty with reading and writing and may copy text without understanding its content. When asked after reading, ‘What was the title?’ they might respond, ‘What was it?’ (P8)

In nonacademic contexts, some students had difficulty performing daily living activities and school‐related self‐care:

He cannot fasten the button on his uniform and requires assistance from his classroom teacher to change clothes during physical education class. (P2)

Unique to the context studied, the participants highlighted students′ restrictions in spiritual occupations, which were deeply linked to the cultural values and norms that shaped the customized school curriculum in Indonesia. These barriers included difficulties executing daily religious routines within the school setting:

When it comes to other non‐academic activities, he is unable to participate fully, including the Dhuha and Dhuhr prayer and reciting the Quran. (P10)

…Student independence is influenced in various ways. It also affects other activities, for example ablution and prayer, as well as extracurricular activities and other non‐academic activity units. (P9)

For example, we have ishoma [an Indonesian acronym for Istirahat, Salat, Makan, which refers to an institutional break time for rest, prayer, and eating], but they are unable to follow the routine. (P6)

## 4. Discussion

The present study sought to describe the experiences of teachers and OTs in identifying activity problems among primary school students with developmental difficulties within the studied context in Indonesia. OTs might address the diverse needs of students in the classroom and help them achieve their full potential by both facilitating desired academic outcomes and compensating for functional learning barriers [25]. Collaboration between teachers and OTs is fundamental to inclusive education [26]; hence, understanding and integrating these distinct perspectives appears crucial for fostering effective interprofessional collaboration. In this study, the perspectives and experiences of OTs and teachers in identifying these activities emerged as two overarching themes: (i) “holistic approaches to the management of student activity problems” and (ii) “students′ functional participation challenges at school.” Within this sample, these themes reflect a structured identification process similar to those observed in well‐resourced settings, such as the private schools on Java island, where most participants worked.

The first theme underscores the importance of an integrated approach to managing student activity problems. Teachers and OTs described how their distinct, yet complementary perspectives shaped the early identification process. Teachers primarily relied on an educational and academic lens, focusing on students′ achievement, classroom engagement, and struggles across multiple subjects. In contrast, OTs identified difficulties through a functional and developmental lens, emphasizing that students′ activity performance, environmental interactions, and developmental patterns fell below age expectations. The participants indicated that this holistic framework required a structured, multistep process backed by effective communication, as reliance on a single perspective might overlook critical student needs. However, the feasibility of such collaboration may depend heavily on systemic factors, including resources, time, and institutional funding [27]. Given that the majority of participants in this study worked in private schools, their experiences likely reflect greater access to structured processes and professional support, suggesting that school type is possibly an important contextual factor shaping both the identification pathway and the quality of support provided. These findings align with Hata et al. [28], who highlighted the challenges of inclusive education in Indonesia and reported that private schools tend to possess greater resources and professional support than their public counterparts. Therefore, although these findings provide a valuable model for systematic identification, they may primarily represent a “best‐case scenario” specific to better‐resourced environments.

In this study, participants noted that teachers initially felt worried or uncertain when working with students with developmental difficulties, underscoring the need for structured training and support systems that embed collaboration as a core practice in school‐based OT. These findings are consistent with previous studies in Indonesia, which have shown that teachers often feel underprepared and lack the competence to identify and support students with developmental difficulties [29–31]. Consultative approaches—including workshops, coaching, and classroom‐based modeling—can address this gap by offering opportunities to exchange practical strategies and strengthen teachers′ capacity [26]. Nevertheless, the transition from teacher uncertainty to effective collaboration appears to rely heavily on structural facilitators that are more readily available in private schools and may be considerably more challenging to achieve in under‐resourced public schools.

The second theme identified in this study highlights the students′ challenges in functional participation at school, reflecting the complex, multifactorial nature of these difficulties. The findings suggest that functional activity problems arise from dynamic interactions among students′ internal barriers, environmental constraints, and specific occupational demands that impede independent participation in school life, rather than reflecting isolated skill deficits. This conceptualization aligns directly with the ICF‐CY′s biopsychosocial model and the OTPF, which elucidate student functioning through dynamic interactions among the individual, the environment, and occupational factors [32–34]. However, in this study, environmental factors were situated in a predominantly private school setting, which often features a greater capacity to recognize and respond to these complex interactions.

Internal barriers—including cognitive, sensory, motor, and social‐emotional dysregulation—appeared to act as core obstacles to students′ functional participation. Our findings suggest that contextual and environmental constraints actively modulate these intrinsic factors. In particular, the availability of social environmental support (from peers and teachers) is a critical external factor that can facilitate or hinder student engagement [35]. Furthermore, home‐based factors (e.g., sleep quality, dietary habits) that affect attention, impulsivity, mood, social skills, executive functioning, and behavior are deeply intertwined with family dynamics, suggesting that family routines and support may shape school participation and serve as important targets for collaborative intervention [36, 37]. This underscores the necessity of adopting an ecological approach that tracks students′ home routines rather than solely focusing on classroom triggers. Notably, the nature of this environmental support appears to depend heavily on the school type and location. Because most participants were from private schools in Java, the reported availability of intensive teacher support and classroom modifications may reflect an optimized scenario. In resource‐constrained environments, a lack of funding and personnel may severely limit the effectiveness of these external facilitators in mitigating students′ intrinsic barriers.

Based on the participants′ workplace experiences, students with developmental difficulties faced restrictions across academic, daily living, and instrumental activities. Consistent with global studies, students with similar challenges commonly experience academic difficulties, particularly in handwriting and literacy [38, 39]. Participants also noted that students exhibited limitations in activities of daily living (ADLs) and self‐care—such as eating, dressing, toileting, maintaining personal hygiene, managing personal belongings, and following school routines. Difficulties in these areas may restrict peer acceptance, reduce social participation, hinder the development of healthy habits, and constrain the self‐efficacy and self‐esteem that support academic performance [40, 41]. Nevertheless, these specific functional restrictions are more likely to be identified in private school settings, where OTs and teachers have greater opportunities to observe nonacademic participation. Thus, the feasibility of implementing holistic evaluations appears closely linked to the resources available in each school setting. These findings underscore the need for assessments and interventions that address both individual capacity and contextual support, particularly in low‐resource settings.

Finally, this study underlines the sociocultural context of participation in Indonesian school settings. Difficulties engaging in religious routines—such as adhering to prayer schedules, performing ablution, and reciting the Quran—were identified as significant challenges in instrumental activities of daily living (IADLs). Currently, the Indonesian curriculum incorporates religious education as a core component aligned with students′ beliefs, with Islamic religious education being heavily emphasized at the primary school level [42, 43]. As religion is deeply embedded in cultural values, participation in these religious activities is widely recognized as contributing to students′ sense of belonging, role fulfillment, and community involvement [44]. Although these spiritual occupations are culturally significant across Indonesia, they may be more readily identified and accommodated as actionable activity problems within private school settings. These findings draw attention to the potential role of school‐based OTs in analyzing how specific cultural and institutional contexts shape students′ meaningful occupations.

### 4.1. Limitations and Future Research

This qualitative study has some limitations. First, the participants worked on Java island, which serves as Indonesia′s administrative and development center and offers greater access to school resources and professional support systems. This concentration limits the transferability of findings to other regions with different access levels. Second, the findings were based on participants′ self‐reported experiences and professional perspectives rather than direct clinical assessment or objective verification of students′ developmental status. Consequently, some examples described by the participants may have involved students who did not have developmental difficulties but were experiencing temporary challenges. To strengthen credibility, shared indicators drawn from previous reports were used in the present study to ensure that the data captured participants′ systemic experiences rather than subjective impressions. Additionally, both teachers and OTs were included to facilitate triangulation of professional perspectives and to increase confidence in the identified activity problems. Third, variation in participants′ years of professional experience may have influenced the depth and nature of the data shared during the FGI sessions. Participants with extensive experience (e.g., greater than 8 years) may have been more familiar with and confident about the topic. In contrast, those with fewer years of experience may have focused more on current challenges and expressed greater uncertainty. To create a comfortable environment for participants to share their perspectives, FGI sessions were organized according to years of work experience. This segmentation captured a multigenerational perspective while respecting variability in professional and clinical reasoning. Fourth, the researchers′ professional backgrounds and experience in clinical and school‐based settings may have influenced their interpretation of the data. Although such expertise could facilitate an in‐depth understanding of functional and developmental issues, it might also introduce professional bias, leading to interpretations framed predominantly through a clinical OT lens rather than analytical neutrality. To address this risk, trustworthiness was ensured in this study by employing multiple qualitative strategies—including triangulation, peer debriefing, member checking, and an audit trail—to maintain the rigor of data collection and analysis. These multiple strategies supported reflexivity, enabled the researchers to challenge their internal assumptions, and ensured that the final themes emerged directly from the participants′ actual words rather than from the researchers′ potential preconceptions. Finally, although the mixed‐profession focus group design enriched interprofessional dialogue by aligning with the study aims, this composition might have constrained the data by potentially directing the dialogue toward an overly harmonious collaborative narrative. Therefore, we recommend that future studies employ profession‐specific focus groups to capture raw, unmediated views. Ultimately, future research is needed to expand the limited Indonesian research base and improve transferability by involving participants from a broader geographic range and ensuring equal representation from public and private schools. Further studies could also focus on developing comprehensive assessment tools suitable for the Indonesian primary school context, as well as effective programs to support all areas of students′ functional participation and to empower teachers and school personnel.

### 4.2. Implication for Occupational Therapy Practice

The implication for occupational therapy practice is listed as follows:

• The findings of this study underscore the dynamic interaction among the multifaceted factors influencing student participation, reinforcing the need for evaluations and interventions that extend beyond students′ individual capacities to encompass the full context of their school environment.
• This study demonstrates that functional participation restrictions extend far beyond traditional academic activities; school‐based practitioners should proactively and comprehensively address these broader nonacademic and IADLs—including self‐care and religious routines—when designing school‐based intervention programs.
• The findings also suggest that holistic approaches to managing student activity problems rely on building an integrated collaborative network among teachers, OTs, school staff, peers, and family members, who collectively shape a supportive ecosystem for the student.
• Although teachers′ initial concerns may not always fall directly within the OT′s primary responsibility, providing systematic consultative and coaching services is highly beneficial. Collaborative consultation may empower teachers and school personnel to implement effective strategies that support students′ participation, ultimately bridging the gap between educational demands and functional capacity in inclusive settings.

## 5. Conclusion

This study described the perspectives and experiences of OTs and teachers in identifying activity problems among primary school students with developmental difficulties in Indonesia, particularly within private school settings. Our findings highlight the value of integrating teacher and OT perspectives to capture the complex, multifactorial nature of students′ functional participation challenges. Although delineating a systematic approach to identification and holistic support, these processes likely reflect the greater availability of resources and professional networks typically associated with private school settings on Java island, where the majority of participants were based. The dynamic interplay between students′ internal functional barriers and environmental constraints—including peer and family support—remains a crucial determinant of school participation. These findings emphasize that holistic approaches to managing restrictions on students′ participation in activities are vital, demonstrating that the roles of teachers and OTs extend far beyond initial identification to directly enabling students to participate fully in everyday school life. However, the institutional capacity to monitor these factors varies considerably across different school types and regions. Ultimately, these findings lay a valuable empirical baseline for future research and the development of comprehensive assessment tools adaptable across diverse educational contexts in Indonesia. School‐based OT practice should proactively expand intervention programs beyond traditional academic programs to include consultative and coaching services, while explicitly acknowledging that such collaborative models require careful systematic adaptation to address the specific resource constraints of Indonesia′s broader educational landscape.

## Author Contributions

P.A. and Y.I. conceptualized and designed the study. P.A., C.R.R., and D.A.N.K. collected the data. P.A., C.R.R., and D.A.N.K. then analyzed and processed the data. P.A. and Y.I. finalized the study results and wrote the initial version of the manuscript. All authors substantially contributed to this study.

## Funding

No funding was received for this manuscript.

## Disclosure

All authors have read and approved the final version of this manuscript.

## Ethics Statement

This study was approved by the Tokyo Metropolitan University Arakawa Campus Research Ethics Committee (Approval No. 24099).

## Consent

All participants provided informed consent prior to their participation.

## Conflicts of Interest

The authors declare no conflicts of interest.

## Data Availability

The data that support the findings of this study were used with permission for the purposes of the current study and are not publicly available. In accordance with institutional guidelines, both digital and physical materials will be permanently disposed of 3 years after publication.
